# Deep Learning with Transformer or Convolutional Neural Network in the Assessment of Tumor-Infiltrating Lymphocytes (TILs) in Breast Cancer Based on US Images: A Dual-Center Retrospective Study

**DOI:** 10.3390/cancers15030838

**Published:** 2023-01-29

**Authors:** Yingying Jia, Ruichao Wu, Xiangyu Lu, Ying Duan, Yangyang Zhu, Yide Ma, Fang Nie

**Affiliations:** 1 Ultrasound Medical Center, Lanzhou University Second Hospital, Cuiyingmen No. 82, Chengguan District, Lanzhou 730030, China; 2 Gansu Province Medical Engineering Research Center for Intelligence Ultrasound, Cuiyingmen No. 82, Chengguan District, Lanzhou 730030, China; 3 Gansu Province Clinical Research Center for Ultrasonography, Cuiyingmen No. 82, Chengguan District, Lanzhou 730030, China; 4 School of Information Science and Engineering, Lanzhou University, No. 222 South Tianshui Road, Lanzhou 730030, China; 5 Department of Ultrasound, Gansu Provincial Cancer Hospital, West Lake East Street No. 2, Qilihe District, Lanzhou 730030, China

**Keywords:** tumor-infiltrating lymphocytes, deep learning, breast neoplasms, ultrasonography

## Abstract

**Simple Summary:**

Tumor-infiltrating lymphocytes (TILs) have been proven to be promising biomarkers associated with therapeutic outcomes and prognosis in breast cancer patients. Increased TIL levels predicted a higher rate of response to neoadjuvant chemotherapy in all molecular subtypes and was also associated with a survival benefit in human epidermal growth factor receptor 2-positive and triple-negative breast cancer. The assessment of TILs was based on surgical pathological sections or needle biopsies; this process was invasive and may have introduced sampling bias in biopsies. Imaging-based biomarkers provide a non-invasive evaluation of TIL levels. The aim of this study was to explore the feasibility of transformer-based or convolutional neural network (CNN)-based deep-learning (DL) models to predict TIL levels in breast cancer from ultrasound (US) images. We confirmed that the ultrasound-based DL approach was a good non-invasive tool for predicting TILs in breast cancer and provided key complementary information in equivocal cases that were prone to sampling bias.

**Abstract:**

This study aimed to explore the feasibility of using a deep-learning (DL) approach to predict TIL levels in breast cancer (BC) from ultrasound (US) images. A total of 494 breast cancer patients with pathologically confirmed invasive BC from two hospitals were retrospectively enrolled. Of these, 396 patients from hospital 1 were divided into the training cohort (*n* = 298) and internal validation (IV) cohort (*n* = 98). Patients from hospital 2 (*n* = 98) were in the external validation (EV) cohort. TIL levels were confirmed by pathological results. Five different DL models were trained for predicting TIL levels in BC using US images from the training cohort and validated on the IV and EV cohorts. The overall best-performing DL model, the attention-based DenseNet121, achieved an AUC of 0.873, an accuracy of 79.5%, a sensitivity of 90.7%, a specificity of 65.9%, and an F1 score of 0.830 in the EV cohort. In addition, the stratified analysis showed that the DL models had good discrimination performance of TIL levels in each of the molecular subgroups. The DL models based on US images of BC patients hold promise for non-invasively predicting TIL levels and helping with individualized treatment decision-making.

## 1. Introduction

In recent years, it has been gradually recognized that the immunogenicity of breast cancer was highly heterogeneous [1], which was reported to be a main factor highly relevant to the therapeutic response and prognosis of BC patients. Tumor-infiltrating lymphocytes (TILs) have been identified as an important immunologic marker that reflects the status of the tumor immune microenvironment [2,3]. Several studies had confirmed that a high TIL level predicted response to neoadjuvant chemotherapy (NAC) in all molecular subtypes and was also associated with a survival benefit in human epidermal growth factor receptor 2(HER2)-positive and triple-negative breast cancer (TNBC). By contrast, increased TILs were an adverse prognostic factor for survival in luminal–HER2-negative breast cancer [4,5].

According to the recommendations by the International TILs Working Group 2014 [6], the standard assessment of TIL levels in breast cancer was based on hematoxylin-eosin (HE) staining of pathological sections of biopsy or resection specimens. As a result, this not only adds to the workload of the pathologists but also to unavoidable subjectivity. On the other hand, this kind of invasive procedure cannot dynamically monitor the changes in the tumor microenvironment. Imaging-based biomarkers hold promise to provide a non-invasive evaluation of TIL levels in BC.

Various studies had explored the association between imaging features and TIL levels in BC, such as ultrasound (US), mammography, and magnetic resonance imaging (MRI) morphological features [7,8,9], quantitative parameters of MRI [10,11,12,13], and 18F-FGD uptake on PET/MRI [14]. However, most of these methods were either subjective or time-consuming. There were also some MRI-based radiomics studies on evaluating TIL levels, although most of these were classical machine-learning methods with limited samples [15].

Compared with MRI, US is a widespread first-line imaging modality used in the diagnosis of breast diseases, given its advantages of low cost, no radiation, portable features, and real-time image acquisition and display. However, ultrasound images have operator-, patient-, and scanner-dependent variations. Meanwhile, the commonly used classical machine-learning methods relying on precise tumor boundaries labeled by radiologists were not that generalized in clinical practice. Deep learning (DL) represented by radiomics can mine high-throughput quantitative features from image data to reveal disease features with the ability to self-learn. Superior to classic ML, the DL approach achieves impressive results and improved robustness in US image analysis by training on large amounts of data [16]. Recent studies have demonstrated that US image-based DL models performed very well in predicting NAC efficacy, axillary lymph node status, molecular subtypes, and risk stratification of breast cancer, etc. [17,18,19,20,21]. To the best of our knowledge, there are no relevant studies applying DL with US images for the prediction of TIL levels in breast cancer.

Hence, we aimed to develop and optimize a novel DL model to predict TIL levels in BC based on US images.

## 2. Materials and Methods

### 2.1. Patients

**Figure 1:**
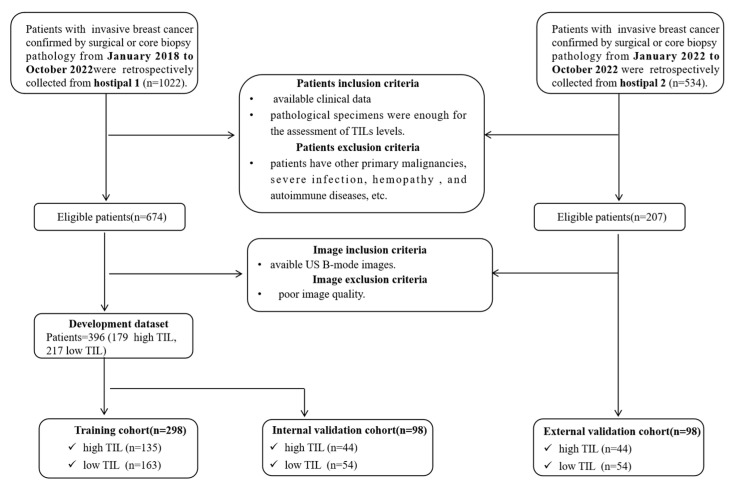
Patient selection flowchart; US, ultrasound; TIL, tumor-infiltrating lymphocyte.

A total of 1022 patients were retrospectively collected from hospital 1 (Lanzhou University Second Hospital) from 1 January 2018, to 31 October 2022, and 534 patients were collected from hospital 2 (Gansu Provincial Cancer Hospital) from 1 January 2022, to 31 October 2022. The inclusion criteria were as follows: (a) patients with invasive breast cancer confirmed by surgical or core biopsy pathology; (b) patients with available US images before any treatment; (c) available clinical data; (d) sufficient pathological specimens for the assessment of TIL levels. The exclusion criteria included: (a) missing important histopathological results; (b) other primary malignancies, severe infection, hemopathy, or autoimmune diseases, etc.; (c) poor US image quality. The flowchart of the patient inclusion process is shown in Figure 1.

### 2.2. Image Acquisition

All the breast US examinations at two hospitals were performed by one of five radiologists with more than five years of US experience using eight different US systems (details of the equipment used in each hospital can be found in Table S1, Supplementary Materials). All US images were acquired 1 or 2 days before performing a biopsy or resection. For patients with more than one breast lesion, the target lesion was defined as the dominant or largest tumor in the affected breast. The target breast lesion was measured at the maximum-diameter plane to determine US size. For consistency, only longitudinal sections of ultrasound images were used.

In this study, two radiologists collaborated to collect the ultrasound images and were prohibited from participating in the subsequent study. Specifically, one radiologist initially collected all ultrasound images of patients eligible for the study based on clinical features and pathological findings. All of these ultrasound data were then handed over to another radiologist, who further screened ultrasound images, focusing only on image quality, without knowing the pathological and clinical information. In this way, poor-quality ultrasound images such as unclear, unstandardized images can be excluded, and the influence of the doctor’s subjective perception and objective ability on the data set during image collection can be effectively avoided.

### 2.3. Clinical and Pathological Analysis

The clinical data were acquired from medical records. Histopathologic data of the breast cancer, including tumor type, histological grade, molecular subtype, estrogen receptor (ER) status, progesterone receptor (PR) status, HER2, and Ki-67 proliferation index, were obtained from pathological reports.

According to the recommendations by the International TILs Working Group 2014 [6], the standard assessment of TIL levels in breast cancer was based on the HE pathological sections of biopsy or resection specimens. TILs include both stromal TILs (sTILs) and intratumoral TILs (iTILs) in tumor tissue. To ensure accuracy and consistency, the recommendations suggested that sTIL levels represent the TIL level of the tumor. The sTIL levels of the breast cancers were defined based on the proportion of the area infiltrated by lymphocytes within the tumor itself plus the adjacent stroma. Consistent with previous studies [7], the TIL levels for this study were categorized as low ≤ 10% and high > 10%. All of the specimens were classified into high and low TIL groups by two pathologists with more than five years of experience who were blinded to the US data.

### 2.4. DL Models

In this study, we used four representative DL networks commonly used in breast US images – ResNet50, DenseNet121, Mobilenet_v3, and Vision Transformer, which were pre-trained with ImageNet (http://www.imagenet.org/, accessed on 1 October 2022). as the basal classification model – to train the DL models based on raw US image data. Furthermore, we developed an attention-based deep-learning model to improve the basic version of DenseNet121. The channel attention and spatial attention module were introduced into DenseNet121 to make the model pay more attention to the information of the area of the tumor. (Detailed in Method S2).

**Figure 2:**
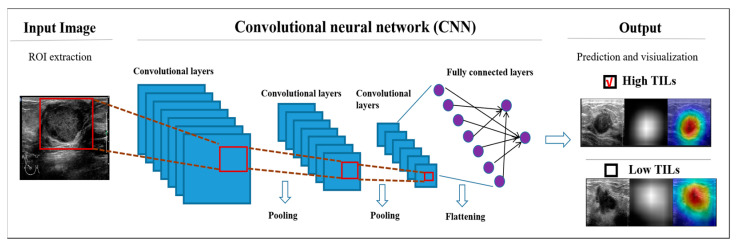
Schematic diagram of the DL models. US, ultrasound; TIL, tumor-infiltrating lymphocyte; ROI, region of interest.

The input of the DL models were US images manually rectangular labeledwith the region of interest (ROI) containing the complete tumor and its border tissue. If posterior and lateral acoustic shadows of the tumor were visible on the US image, the ROI also needed to include part of it. The DL algorithm is capable of learning hierarchical representations from the raw US imaging data provided as input. After sequential activation of the convolution and pooling layers, the DL model output the probability of TIL levels (Figure 2).

Due to the limited training data in our dataset, we used data augmentation for image augmentation. Data augmentation included flipping, scaling, rotating, and contrast changes. All images were resized to 224 × 224 pixels to standardize the distance scale. Additionally, data augmentation strategies have been shown to prevent neural network overfitting. During training, the network was iteratively trained using the binary cross-entropy loss function for a total of 60 epochs. When iterating to 40 epochs, we selected the model with the best AUC in the last 20 epochs. To improve the reliability of the network, each network was trained five times and the model with the median result was chosen for comparison. (Details of the methods, including data preprocessing, the structure of the models, the strategy for training the models, and measuring the performance of the models, are shown in Methods S1–S4.).

To better interpret the model diagnosis process, we used the method of gradient-weighted class activation mapping (Grad-CAM) [22] to produce heat maps to display the pixels in the ROIs that provide the greatest contribution to the classification output.

### 2.5. Stratified Analysis to Assess the Diagnostic Value

Increased TIL levels have different results on the prognosis in different BC molecular subtypes. We further performed a stratified analysis in the EV cohort to verify the diagnostic power of the attention-based DenseNet121 model. Patients were stratified into four subgroups according to molecular subtypes, including HR+ and HER2−, HR+ and HER2+, ER−, PR− and HER2+, and triple-negative subgroups.

### 2.6. Statistical Analysis

Statistical analysis was performed using SPSS 26.0 (IBM Corp., Armonk, NY, USA) and Python 3.6. Continuous variables were described as means ± standard deviations (SDs) and comparisons between two groups were made using the Mann–Whitney U test or student’s *t*-test. Categorical variables were expressed as numbers and percentages, and comparisons between two groups were made using the chi-squared test or Fisher’s exact test. Receiver operating characteristic (ROC) curve analysis was used to evaluate the diagnostic performance of the model, and areas under the ROC curve (AUCs) were calculated with 95% confidence intervals (CIs). A precision–recall (P-R) curve was plotted to evaluate the accuracy of the model, and the F1 score was calculated with 95% CIs. The accuracy, sensitivity, specificity, positive predictive value (PPV), and negative predictive value (NPV) with 95% CIs were reported for the DL models. All statistical analyses were two-sided, and the statistical significance was set at *p* < 0.05. The number of true-positive, false-positive, true-negative, and false-negative findings of the models on validation cohorts was described in a 2 × 2 contingency table representing the confusion matrix.

## 3. Results

### 3.1. Baseline Characters

A total of 494 breast cancer patients with 494 lesions were ultimately enrolled in this dual-center study (Figure 1). A total of 396 patients from hospital 1 were used as the main cohort to reduce overfitting or bias in the study. Among these, 298 patients from hospital 1 collected before 2022 were divided into the training cohort for model development, while 98 patients from 2022 were used as the IV cohort to simulate prospective experimental conditions. Patients from hospital 2 (*n* = 98) collected in 2022 were in the EV cohort. The clinical-pathological characteristics of the patients were described in Table 1. There were no significant differences in the clinical-pathological characteristics between the training cohort and the two validation cohorts (*p* > 0.05).

### 3.2. Performance of DL Models

**Figure 3:**
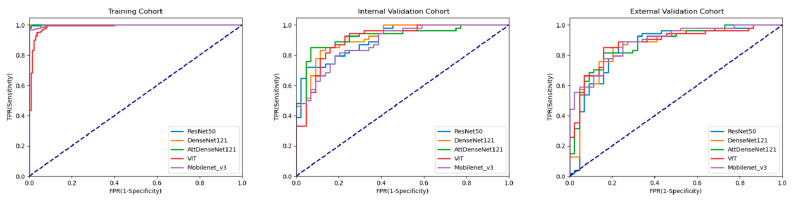
Comparison of ROC curves among DL models for predicting TIL levels in the training cohort, internal validation, and external validation cohort. AttDenseNet121 attention-based DenseNet121, VIT Vision Transformer.

**Figure 4:**
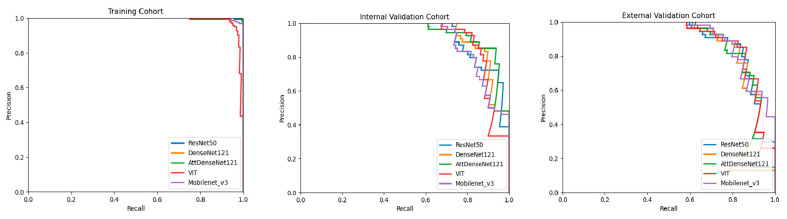
Comparison of P-R curves among DL models for predicting TIL levels in the training cohort, internal validation cohort, and external validation cohort. AttDenseNet121 attention-based DenseNet121, VIT Vision Transformer.

All five DL models performed well in predicting TIL levels based on breast cancer US images. In IV cohorts, the AUCs were 0.906 (95% CI: 0.831, 0.956) for the ResNet50 model, 0.919 (95% CI: 0.847, 0.965) for the DenseNet121 model, 0.922 (95% CI: 0.850, 0.967) for the attention-based DenseNet121 model, 0.885 (95% CI: 0.805, 0.941) for the Mobilenet_v3 model, and 0.907 (95% CI: 0.832, 0.957) for the Vision Transformer model. The F1 scores were 0.824 (95% CI: 0.754, 0.895) for the ResNet50 model, 0.846 (95% CI: 0.779, 0.915) for the DenseNet121 model, 0.851 (0.784,0.919) for the attention-based DenseNet121 model, 0.811 (95% CI: 0.736, 0.887) for the Mobilenet_v3 model, and 0.862 (95% CI: 0.797, 0.927) for the Vision Transformer model. For the EV cohort, the AUCs were 0.858 (95% CI: 0.774, 0.921) for the ResNet50 model, 0.867 (95% CI: 0.784, 0.927) for the DenseNet121 model, 0.873 (95% CI: 0.791, 0.932) for the attention-based DenseNet121 model, 0.888 (95% CI: 0.808, 0.947) for the Mobilenet_v3 model, and 0.878 (95% CI: 0.796, 0.935) for the Vision Transformer model. The F1 scores were 0.836 (95% CI: 0.766, 0.906) for the ResNet50 model, 0.844 (95% CI: 0.775, 0.912) for the DenseNet121 model, 0.830 (95% CI: 0.762, 0.898) for the attention-based DenseNet121 model, 0.803 (95% CI: 0.726, 0.881) for the Mobilenet_v3 model, and 0.844 (95% CI: 0.775, 0.913) for the Vision Transformer model. The ROC curve was plotted to demonstrate the comparative results of AUCs in Figure 3. The P-R curve was plotted to demonstrate the relationship between the precision and recall rate of different models in Figure 4. Compared with the DenseNet121 model, the attention-based DenseNet121 model achieved better results (Table 2).

For the IV cohort, the accuracies were 79.5% for the ResNet50 model, 82.8% for the DenseNet121 model, 83.6% for the attention-based DenseNet121 model, 79.6% for the Mobilenet_v3 model, and 84.7% for the Vision Transformer model. The sensitivities were 87.0% (95% CI: 77.8%, 96.2%), 87.0% (95% CI: 77.8%, 96.2%), 85.2% (95% CI: 75.5%, 94.9%), 79.6% (95% CI: 68.6%, 90.6%), and 87.0% (95% CI: 77.8%, 96.2%); and the specificities were 70.4% (95% CI: 56.5%, 84.3%), 77.2% (95% CI: 64.5%, 90.0%), 81.8% (95% CI: 70.1%, 93.5%), 79.5% (95% CI: 67.3%, 91.8%), and 81.8% (95% CI: 70.1, 93.5%), respectively. For the EV cohort, the accuracies were 81.6% for the ResNet50 model, 81.6% for the DenseNet121 model, 79.5% for the attention-based DenseNet121 model, 79.6% for the Mobilenet_v3 model, and 82.7% for the Vision Transformer model.; the sensitivities were 85.1% (95% CI: 75.4%, 94.8%), 85.1% (95% CI: 75.4%,94.8%), 90.7% (95% CI: 82.8%, 98.6%), 75.9% (95% CI: 64.3%,87.5%), and 85.2% (95% CI: 75.5%, 94.9%); and the specificities were 77.2% (95% CI: 64.5%, 90.0%), 79.5% (95% CI: 67.2%, 91.8%), 65.9% (95% CI: 51.5%,80.3%), 84.1% (95% CI: 73.0%, 95.2%), and 79.5% (95% CI: 67.3%, 91.8%), respectively. The classification confusion matrices that report the number of true-positive, false-positive, true-negative, and false-negative results for the attention-based DenseNet121 DL model in validation cohorts was shown in Table 3.

Others tumor types included invasive lobular carcinoma, intraductal papillary carcinoma, and mucinous carcinoma. P1 indicates the significance between the training and the IV cohort; P2 indicates the significance between the training and the EV cohort. Abbreviations: IV, internal validation; EV, external validation; ER, estrogen receptor; PR, progesterone receptor; HR, hormone receptor; HER2, human epidermal growth factor receptor 2.

**Figure 5:**
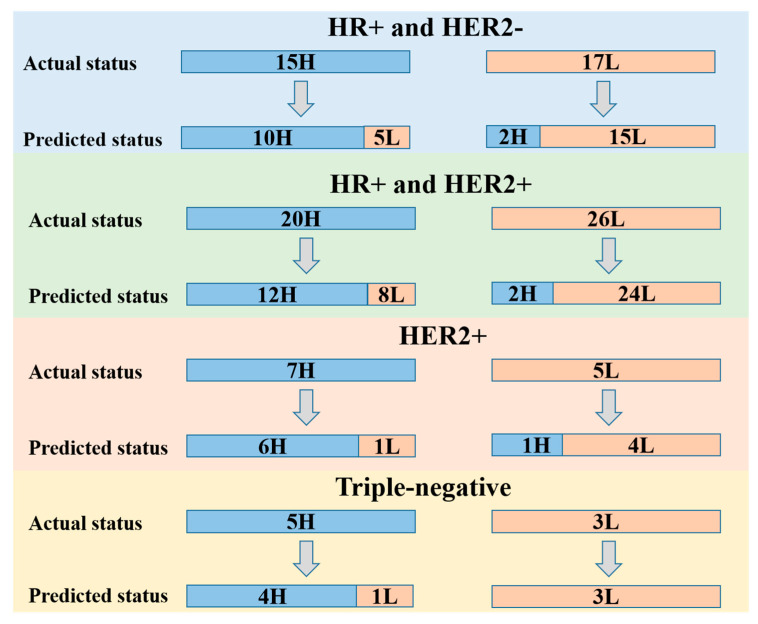
Stratified performance of the attention-based DenseNet121 DL model based on breast cancer molecular subtypes including HR+ and HER2−, HR+ and HER2+, ER−, PR− and HER2+, and triple-negative subgroups. H, high TIL level; L, low TIL level; ER, estrogen receptor; PR, progesterone receptor; HER2, human epidermal growth factor receptor 2; HR, hormone receptor.

Furthermore, for the EV cohort, we conducted a stratified analysis of the performance of the attention-based DenseNet121 DL model based on four different molecular subtypes of BC (Figure 5). The prediction performance of the DL model in each of the HR+ and HER2−, HR+ and HER2−, ER−, PR−, and HER2+, and triple-negative subgroups are shown in Table 4.

### 3.3. Visual Interpretation of the Model

**Figure 6:**
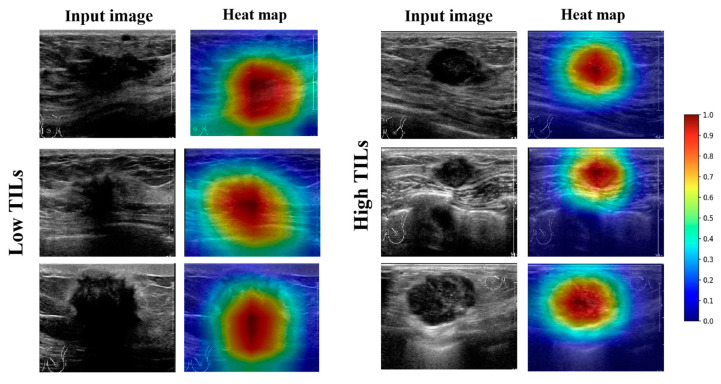
Examples of high-TIL-level and low-TIL-level tumors with B-mode US images and corresponding heat maps generated by CLA-HDM. The heat maps showed that the interior of the tumor is valuable for predicting high TIL levels, while the tumor interior and posterior predict low TIL levels. TILs, tumor-infiltrating lymphocytes.

Heat maps were used to visually interpret the DL model’s decision-making. Two groups of heat maps for the attention-based Densenet121DL model are shown in Figure 6 as examples. The DL model provided accurate diagnostic outcomes, with the heat maps illustrating distinguishable color patterns. The red parts of the map indicate the area contributing more information to the network’s diagnostic process. By screening all heat maps, we found different common patterns in high and low-TIL-level tumors. In most US images of high-TIL-level tumors, the valuable area often tends to cluster on the interior of the tumor, followed by margin features. In addition, in most US images of low-TIL-level tumors, the valuable area often tended to cluster on the interior and posterior of the tumors. To some extent, this may explain the discrimination ability of the DL model, which is consistent with previous clinical studies.

## 4. Discussion

TILs have emerged as clinically relevant and reproducible biomarkers with predictive significance for therapeutic efficacy and prognosis in BC patients [3]. Given this importance, the St Gallen Consensus Conference, WHO, and ESMO 2019 Guidelines all recommend that pathological evaluation should include TIL quantification and reporting in TNBC and HER2+ BC [23,24]. However, the main factor limiting widespread use of TILs in clinical practice was their invasive nature. Continuous studies were therefore carried out on a non-invasive method of accurately predicting TIL levels in BC [25].

Initially, there were several studies investigating the relationship between imaging features and TIL levels in BC. Fukui et al. reported that more lobulated margin, weaker internal echo level, and enhanced posterior echoes were predictors of lymphocyte-predominant breast cancer [26]. Furthermore, another study revealed that TNBC tumors with high TIL levels were more likely to have oval/round shapes, circumscribed or microlobulated margins, and enhanced posterior echoes [8]. Although the findings of these studies revealed that the imaging features had the potential to predict TIL levels, these findings were operator-dependent with lower repeatability. Subsequently, there were also some MRI-based radiomics studies about evaluating TIL levels in BC [15,27,28]; most of these studies used a classic machine-learning (ML) approach with a small sample size in a single center. All these factors seriously affected the accuracy and generalization of the model [16]. More recently, the deep-learning approach has made substantial progress with unsupervised learning. It can avoid the influence of subjective factors and achieve a more accurate result at a faster speed. To our knowledge, this is the first study applying the DL approach with US images for predicting TIL levels in BC from dual centers. A total of 494 patients from two hospitals participated in this study, ensuring the credibility of the study and providing a good basis for future studies with a larger sample size.

In this study, the five DL models all performed well in predicting the TIL level of BC. Compared with classical machine-learning methods, the five DL models all use a large number of convolution kernels for feature extraction, which can extract advanced semantic information to assist evaluation. ResNet50 is based on VGG11 and introduces a skip connection layer for residual learning. The residual structure ensures the integrity of information and avoids gradient disappearance or gradient explosion. MobileNet_v3 is a lightweight deep neural network that mainly uses depthwise separable convolutions, inverted residuals, attention mechanisms, and linear bottlenecks. These modules greatly reduce the number of computational parameters while ensuring the performance of the network. Because MobileNet_v3 does not require high device performance, it can be widely used in US images. The MobileNet_v3 DL mode had a higher AUC value than the ResNet50 DL model in the IV cohort and a lower AUC value in the EV cohort. The overall performance of the ResNet50 and MobileNet_v3 DL models was similar. Vision Transformer is a model that applies Transformer to image classification. When there is enough data for pre-training, the performance of Vision Transformer may exceed that of CNN. In this study, Vision Transformer thus performed better than the two CNN models indicated above.

DenseNet uses a more aggressive dense connection mechanism than ResNet. Each layer is connected to each other, so that the network does not completely rely on the features of the upper layer for extraction, making the reuse and extraction of features more accurate [29]. Therefore, DenseNet has very good anti-overfitting performance, making it especially suitable for applications where training data are relatively scarce. Our results show that the overall performance of Densenet121 was better than that of the ResNet50 and Mobilenet_v3 DL models. The attention-based DenseNet121 DL model we proposed was to further improve the basic version of DenseNet121. The advantage of attention-based DL models was that the model with the added attention module paid more attention to the features in the tumor area and filtered out useless peripheral information [30]. The combination of channel attention and spatial attention modules can transform various deformation data in space and automatically capture important regional features. The overall performance of the attention-based DenseNet121 DL model was thus better than the basic DenseNet121.

Furthermore, the stratified analysis in the EV cohort according to the molecular subtypes also showed good performance. The value of TIL levels in HER2+ and triple-negative breast cancer has been widely recognized. Specifically, our DL model performed better in predicting TIL levels in HER2+ and triple-negative breast cancer. In HR+ and HER2+ subtype breast cancer, the DL model had a higher false negative rate in predicting high TILs. This means that in the HR+ and HER2+ subtypes, part of the low-TIL tumors had some imaging features that overlapped with those of high-TIL tumors.

The DL model not only provided a clinical judgment of TIL levels in BC, but also visualized its decision-making by heat maps. There were different color patterns between the heat maps of high- and low-TIL tumors. To some extent, this may explain the discrimination ability of the DL models; it is also consistent with the result of previous clinical studies [8,31]. The US image features of BC were strongly associated with organizational construction. In low-TIL tumors, fibrosis increased in tumor stromal and the posterior echoes were often attenuated [32]. The attenuated posterior echoes were a typical feature of low-TIL tumors. The DL models paid more attention to the interior and posterior of the lesion on US images. In high-TIL tumors, the tumor tissues rich in water-soluble components have less attenuation; as a result, the internal echo was lower, and posterior echoes were enhanced. As the internal organizational construction was different from low-TIL tumors, the DL model paid more attention to the interior of the tumor, followed by margin features. However, unlike low-TIL images, the model does not focus on the posterior features in high-TIL ultrasound images. It may be that internal features of the lesions contribute more to the diagnostic process. Even so, the relationship between image features and pathological characteristics still needs direct evidence for confirmation. In any event, the highlighted regions in the heat maps were helpful to identify the representative characteristics of high- and low-TIL tumors.

Compared with other studies on predicting TIL levels in BC using medical imaging methods, our study took a more objective approach—DL with Transformer or Convolutional Neural Network—and the models were trained and validated by a larger dataset of standardized US images from dual centers. Compared with MRI, US had the advantage of lower cost, simplicity, and greater availability with tremendous clinical potential and economic benefits. More importantly, it was proved that the ultrasound-based DL approach was a good non-invasive tool for predicting TILs in BC and providing key complementary information in equivocal cases that are prone to sampling bias.

There are several limitations to this study. Firstly, this was a retrospective study resulting in inevitable bias. Although our study involved dual center databases, more prospective cohorts were needed to further validate the generalization ability of the model. Secondly, although all US examinations were performed by experienced physicians in a standardized way, there were still some variabilities in the quality of the images performed by multiple physicians. Thirdly, as a common problem with many other DL models, the biological mechanism of how the DL approach accurately differentiates high and low TIL levels cannot be interpreted exactly.

## 5. Conclusions

In conclusion, we demonstrated that our DL models based on US images perform satisfactorily in predicting TIL levels. The overall best-performing DL model reached an AUC of 0.873, an accuracy of 79.5%, a sensitivity of 90.7%, a specificity of 65.9%, and an F1 score of 0.830. With further validation in a larger sample size from more centers, the DL approach has great potential to serve as a non-invasive tool to predict TIL levels and make the management of patients becomes more precise.

## Figures and Tables

**Table 1:** The clinical-pathological characteristics of the patients.

Characteristics	Training Cohort (*n* = 298)	IV Cohort (*n* = 98)	*P*1	EV Cohort (*n* = 98)	*P*2
Age, years, mean ± SD	53.1 ± 11.9	52.4 ± 11.8	0.76	54.1 ± 10.7	0.13
US size, cm mean ± SD	2.38 ± 0.97	2.44 ± 0.91	0.50	2.50 ± 1.01	0.57
Ki67			0.32		
≤20	103 (34.6%)	28 (28.6%)		36 (36.7%)	0.72
>20	195 (65.4%)	70 (71.4%)		62 (63.3%)	
ER			0.61		0.30
Positive	207 (69.5%)	71 (72.4%)		74 (75.5%)	
Negative	91 (30.5%)	27 (27.6%)		24 (24.5%)	
PR			0.82		0.52
Positive	166 (55.7%)	53 (54.1%)		55 (56.1%)	
Negative	132 (44.3%)	45 (45.9%)		43 (43.9%)	
HER2			0.52		0.72
Positive	172 (57.7%)	57 (58.2%)		59 (60.2%)	
Negative	126 (42.3%)	41 (41.8%)		39 (39.8%)	
Molecular subtype			0.63		0.99
HR+ and HER2−	101 (33.9%)	33 (33.7%)		32 (32.7%)	
HR+ and HER2+	138 (46.3%)	42 (42.9%)		46 (46.9%)	
HER2+	34 (11.4%)	16 (16.3%)		12 (12.2%)	
Triple-negative	25 (8.4%)	7 (7.1%)		8 (8.2%)	
Histological grade			0.24		0.21
1	14 (4.7%)	5 (5.1%)		6 (5.1%)	
2	280(94.0%)	89 (90.8%)		88 (89.8%)	
3	4 (1.3%)	4 (4.1%)		4 (4.1%)	
Tumor type			0.43		0.68
Invasive ductal carcinoma	283 (95.0%)	91 (92.9%)		92 (93.9%)	
Others	15 (5.0%)	7 (7.1%)		6 (6.1%)	

**Table 2:** Performance of five DL Models according to validation cohorts.

	ResNet50	DenseNet121	Attention-Based DenseNet121	Mobilenet_v3	Vision Transformer
**IV cohort (*n* = 98)**					
AUC	0.906 [0.831, 0.956]	0.919 [0.847, 0.965]	0.922 [0.850, 0.967]	0.885 [0.805, 0.941]	0.907 [0.832, 0.957]
ACC(%)	79.5 [71.5, 87.6]	82.8 [75.0, 90.2]	83.6 [76.3, 91.0]	79.6 [71.5, 87.6]	84.7 [77.4, 91.9]
SENS(%)	87.0 [77.8, 96.2]	87.0 [77.8, 96.2]	85.2 [75.5, 94.9]	79.6 [68.6, 90.6]	87.0 [77.8, 96.2]
SPEC(%)	70.4 [56.5, 84.3]	77.2 [64.5, 90.0]	81.8 [70.1, 93.5]	79.5 [67.3, 91.8]	81.8 [70.1, 93.5]
PPV(%)	78.3 [67.6, 88.9]	82.4 [72.3, 92.5]	85.2 [75.5, 94.9]	82.7 [72.2, 93.2]	85.5 [75.9, 95.0]
NPV(%)	81.5 [68.8, 82.4]	82.9 [71.0, 94.8]	81.8 [70.1, 93.5]	76.1 [63.4, 88.7]	83.7 [72.4, 95.1]
F1 score	0.824 [0.754, 0.895]	0.846 [0.779, 0.915]	0.851 [0.784, 0.919]	0.811 [0.736, 0.887]	0.862 [0.797, 0.927]
**EV cohort (*n* = 98)**					
AUC	0.858 [0.774, 0.921]	0.867 [0.784, 0.927]	0.873 [0.791, 0.932]	0.888 [0.808, 0.947]	0.878 [0.796, 0.935]
ACC(%)	81.6 [73.8, 89.3]	81.6 [75.0, 90.2]	79.5 [71.5, 87.6]	79.6 [71.5, 87.7]	82.7 [75.1, 90.2]
SENS(%)	85.1 [75.4, 94.8]	85.1 [75.4, 94.8]	90.7 [82.8, 98.6]	75.9 [64.3, 87.5]	85.2 [75.5, 94.9]
SPEC(%)	77.2 [64.5, 90.0]	79.5 [67.2, 91.8]	65.9 [51.5, 80.3]	84.1 [73.0, 95.2]	79.5 [67.3, 91.8]
PPV(%)	82.1 [71.8, 92.3]	83.6 [73.6, 93.6]	76.6 [65.9, 87.1]	85.4 [75.2, 95.7]	83.6 [73.6, 93.6]
NPV(%)	80.9 [68.7, 93.1]	81.3 [69.4, 93.3]	85.3 [72.9, 97.6]	74.0 [61.5, 86.5]	81.3 [69.4, 93.4]
F1 score	0.836 [0.766, 0.906]	0.844 [0.775, 0.912]	0.830 [0.762, 0.898]	0.803 [0.726, 0.881]	0.844 [0.775, 0.913]

95% confidence intervals are included in brackets. Abbreviations: AUC—area under the receiver operating characteristic curve, ACC—accuracy, SENS—sensitivity, SPEC—specificity, PPV—positive predict value, NPV—negative predict value, IV—internal validation, EV—external validation.

**Table 3:** Confusion Matrices for attention-based DenseNet121 DL model according to Validation cohorts.

	ResNet50 (Truth)		DenseNet121 (Truth)		Att DenseNet121 (Truth)		MobileNet_v3 (Truth)		Vision Transformer (Truth)	
Prediction	High	Low	High	Low	High	Low	High	Low		
IV cohort										
High	31	7	34	7	36	8	35	11	36	7
Low	13	47	10	47	8	46	9	43	8	47
EV cohort										
High	34	8	35	8	29	5	37	13	35	8
Low	10	46	9	46	15	49	7	41	9	46

**Table 4:** Stratified performance of the attention-based DenseNet121 DL model based on breast cancer molecular subtypes in EV cohort.

Molecular Subtypes	ACC	SENS	SPEC	PPV	NPV
HR+ and HER2−	78.1%	75.0%	83.3%	88.2%	66.7%
HR+ and HER2+	78.3%	75.0%	85.7%	66.7%	60.0%
ER-, PR- and HER2+	83.3%	80.0%	85.7%	80.0%	85.7%
Triple-negative	87.5%	75.0%	100.0%	100.0%	80.0%

Abbreviations: ER, estrogen receptor; PR, progesterone receptor; HR, hormone receptor; HER2, human epidermal growth factor receptor 2; ACC, accuracy; SENS, sensitivity; SPEC, specificity; PPV, positive predict value; NPV, negative predict value.

## Data Availability

Clinical and ultrasound images are not public to protect patient privacy. Original images may be available upon reasonable request to the corresponding authors (F.N. and Y.M.). The code is available at https://github.com/wrc0616/breast, accessed on 4 December 2022).

## Supplementary Materials

The following supporting information can be downloaded at https://www.mdpi.com/article/10.3390/cancers15030838/s1: Methods S1: Data preprocessing; Methods S2: Structure of our model; Methods S3: Strategy of training our model; Methods S4: Measuring the performance of our model; Table S1: Details of the equipment used in each hospital. References [33,34,35,36,37,38,39,40,41,42,43,44,45,46,47,48,49,50,51,52] are cited in the supplementary materials.
