# Supplementary material for: Deep Learning with Transformer or Convolutional Neural Network in the Assessment of Tumor-Infiltrating Lymphocytes (TILs) in Breast Cancer Based on US Images: A Dual-Center Retrospective Study
Source: Cancers (Basel). 2023 Jan 29;15(3):838. doi: 10.3390/cancers15030838 (PMC9913836; doi:10.3390/cancers15030838)
Supplement: Supplementary file 1 [file cancers-15-00838-s001.zip › cancers-2109523-supplementary.pdf]

Supplementary Material

Methods

Method S1. Data preprocessing.

Method S2. Structure of our model.

Method S3. Strategy of training our model.

Method S4. Measuring the performance of our model.

Figures

Fig. S1. Overall architecture of the ResNet50 model

Fig. S2. Overall architecture of the MobileNet_v3 model

Fig. S3. Overall architecture of the Vision Transformer model

Fig. S4. Overall architecture of the DenseNet121 model

Fig. S5. Overall architecture of the attention-based DenseNet121 model

Tables

Table S1. Detailed make and model of ultrasound diagnostic instrument used in the study.

References

Methods S1. Data preprocessing

Data preprocessing

In deep-learning, prior knowledge refers to integrating our cognition of a specific task into the design or training of the model so that the model can be trained spontaneously in the desired direction. In this study, we used B-mode ultrasound (BUS) to predict high and low TILs levels.

The data from the two hospitals were all preprocessed. Before feeding forward into the model, all inputs were standardized to a mean of 0 and a variance of 1, which is a common processing method for image data in deep-learning. The formula for standardizing image data is as follows:

$$I_i'(\mathrm{x},\mathrm{y}) = \frac{I_i(x,y) - \mu}{\sigma}$$

where $I_i(x,y)$ denotes the pixel value of the original image I_i at (x, y) (normalized to [0, 1]), $I_i'(\mathrm{x},\mathrm{y})$ denotes the pixel value of the standardized image I_i' at (x, y). μ denotes the mean value, and σ denotes the variance of the entire image following the formula below:

$$\mu = \frac{1}{K}\sum_{k=1}^{K}\frac{1}{M \times N}\sum_{x=0}^{M-1}\sum_{y=0}^{N-1} I_k(x,y)$$

$$\sigma^2 = \frac{1}{K}\sum_{k=1}^{K}\frac{1}{M \times N}\sum_{x=0}^{M-1}\sum_{y=0}^{N-1} (I_k(x,y) - \mu)^2$$

where K denotes the number of training images. The above standardization method was employed for each channel of inputs.

Data augmentation[1] is another common method to increase the size of the dataset so that the model can learn more features with invariant characteristics to prevent the occurrence of overfitting, particularly in few-shot learning. Commonly used data

augmentation methods include random vertical and horizontal flipping, random rotation, and random cropping. To get the most out of the data samples, we use all the data augmentation methods. In this study, we set the vertical and horizontal flipping probabilities to 0.5, and the random rotation range was [-90°, 90°]. In other words, the input of the model will flip vertically and horizontally with a probability of 0.5, and rotate an angle randomly within the range of -90°to 90°. Finally, we resize all inputs to (256, 256) and center crop to (224, 224) so that we can train our models in parallel for better performance.

2D convolution

The convolution[2] has been proven to be one of the most effective tools for extracting features of high-dimensional data, particularly local features. Through layer-by-layer stacking of the convolutional layer, high-level semantic features can be effectively extracted. The convolution formula is as follows:

$$\mathrm{f(x,y)} = \sum_{m=-k_w}^{k_w} \sum_{n=-k_h}^{k_h} I(x+m, y+n)k(m,n)$$

where I denotes the input image and f is the output image (also called the feature map). k is the learnable convolution kernel, and k_w and k_h are the width and height of k.

In this study, the dataset is a 2D image, so we use a large number of 2D convolutional networks. There are a large number of convolution operations in the 4 DCNN models which we use.

Rectified linear unit

To imitate the work of brain neurons and enhance the model's ability to fit nonlinear functions, an activation function usually follows each layer in deep models. The rectified

linear unit (ReLU)[3] is a commonly used activation function. The mathematical definition of ReLU is as follows:

$$\mathrm{ReLU(x)} = \max(0, x)$$

The biggest characteristic of ReLU is that it not only introduces nonlinearity but also maintains a gradient of 1 in the interval of $x > 0$, which effectively avoids the phenomenon of gradient explosion and gradient disappearance in other activation functions such as sigmoid.

In our study, we applied ReLU as the activation function of each layer, except for the last layer. In the last layer, we applied the Sigmoid function as an activation function to output probabilities. The mathematical definition of Sigmoid is as follows:

$$\mathrm{Sigmoid(x)} = \frac{1}{1 + e^{-x}}$$

Batch normalization

Batch normalization (BN)[4] is used to solve the difficulty of training caused by the continuous change in the distribution parameters of the data during the training process in DL. As mentioned above, we standardized the inputs to mean 0 and variance 1. However, during training, the parameters of the model change. For a certain layer, the distribution of inputs captured by the model in the last training epoch may not be consistent with that in the next training epoch. Changing the data distribution increases the difficulty of training. Owing to the usage of the GPU, we trained the model in parallel with multiple inputs, called mini-batch, to accelerate the training procedure. We used mini-batch to continuously update the distribution parameters of inputs and standardize inputs to mean 0 and variance 1. Specifically, in the training phase, we calculated the

mean and variance of the data in the minibatch according to the following formula and updated the total mean and total variance.

$$\mu_B = \frac{1}{m}\sum_{k=1}^{m} x_k$$

$$\sigma^2 = \frac{1}{m}\sum_{k=1}^{m} (x_k - \mu_B)^2$$

Then, we standardize the inputs:

$$\hat{x} = \frac{x - \mu_B}{\sqrt{\sigma^2 + \epsilon}}$$

where ϵ is a small number used to stabilize the output. For the model to learn a distribution that is most conducive to subsequent tasks, let γ and β be learnable parameters, and the final output is:

$$y = \gamma\,\hat{x} + \beta$$

In this study, we followed the ResNet-50 structure and placed BN after each convolution layer.

Global average pooling

Global average pooling (GAP)[5] is one of the methods used to aggregate global features in deep-learning models. Usually, aggregating global features can be achieved by global average pooling and global maximum pooling. As the name suggests, the global average pooling averages over global features:

$$\text{GAP}(f) = \frac{1}{M}\sum_{x} f(x)$$

where $f(x)$ denotes a feature map while M is the number of features in $f(x)$.

Fully connected

In classification or regression tasks, the fully connected layer (FC) can usually act as

an output layer at the end of the model[6]. In this study, we apply the FC layer to the classification head to obtain the level of TIL.

Attention mechanism

Attention is a complex cognitive function that is indispensable for human beings. One important property of perception is that humans do not tend to process whole information in its entirety at once. Instead, humans tend to selectively concentrate on a part of the information when and where it is needed, but ignore other perceivable information at the same time. For instance, humans usually don't see all the scenes from the beginning to the end when visually perceiving things, but instead, observe and pay attention to specific parts as needed. When humans find that a scene often has something they want to observe in a certain part, they will learn to focus on that part when similar scenes appear again and focus more attention on the useful part. This is a means for humans to quickly select high-value information from massive information using limited processing resources. The attention mechanism greatly improves the efficiency and accuracy of perceptual information processing.

The attention mechanism[7, 8] of humans can be divided into two categories according to its generation manner. The first category is the bottom-up unconscious attention, called saliency-based attention, which is driven by external stimuli. For example, people are more likely to hear loud voices during a conversation. It is similar to the max-pooling and gating mechanism in deep learning, which passes more appropriate values (i.e., larger values) to the next step. The second category is top-down conscious attention, called focused attention. Focused attention refers to the attention that has a

predetermined purpose and relies on specific tasks. It enables humans to focus attention on a certain object consciously and actively. Most of the attention mechanisms in deep learning are designed according to specific tasks so that most of them are focused attention.

As mentioned above, attention mechanism can be used as a resource allocation scheme, which is the main means to solve the problem of information overload. In the case of limited computing power, it can process more important information with limited computing resources. Hence, some researchers bring attention to the computer vision area. proposed a saliency-based visual attention model that extracts local low-level visual features to get some potential salient regions.

Methods S2. Structure of our models

The five DL models all use a large number of convolution kernels for feature extraction, which can extract advanced semantic information to assist evaluation. ResNet50[9] is based on VGG11[10] and introduces a skip connection layer for residual learning. The residual structure ensures the integrity of information and avoids gradient disappearance or gradient explosion, which makes deep networks unable to train. Resnet50 works well in predicting the level of TIL of BC (**Fig. S1**). MobileNet_v3[11] is a lightweight deep neural network that mainly uses depthwise separable convolutions, inverted residuals, attention mechanisms, and linear bottlenecks. These modules greatly reduce the number of computational parameters while ensuring the performance of the network. Therefore, MobileNet_v3 has a fast prediction speed in predicting the TIL level of BC and does not require high device performance, so it can be widely used in medical testing (**Fig. S2**). Vision Transformer[12] is a model that applies Transformer to image

classification. When there is enough data for pre-training, the performance of Vision Transformer will exceed CNN. It breaks through the limitation of Transformer's lack of inductive bias, and can get better results in downstream tasks (**Fig. S3**). DenseNet[13] uses a more aggressive dense connection mechanism than ResNet. Each layer is connected to each other, so that the network does not completely rely on the features of the upper layer for extraction, making the reuse and extraction of features more accurate. Therefore, DenseNet has very good anti-overfitting performance, especially suitable for applications where training data is relatively scarce. The experimental results show that Densenet121 has a good predictive effect in predicting the TIL level of BC (**Fig. S4**). The Attention-based DenseNet121 network we proposed is improved on the basis of DenseNet121. We introduced the attention module to make the network pay more attention to the information around the tumor. The attention module mainly consists of two parts: channel attention[14] and spatial attention[15]. Channel attention first performs channel compression of global spatial information on the feature layer extracted by the network, then performs feature learning on the channel dimension to form the importance of each different channel, and finally assigns different weights to each channel through the Sigmoid activation function. Spatial attention is to perform global pooling and average pooling on the feature layers extracted by the network, merge them into a feature map with a dimension of two, and then perform a convolution operation to generate a 1-dimensional feature map, and finally use the Sigmoid activation function to assign different weights. The combination of two attention modules can transform various deformation data in space and automatically capture

important regional features, which play an important role in predicting the TIL level of BC (**Fig. S5**).

Our proposed attention module, where the channel attention module can be defined as:

$$M_c(F) = \sigma\left(MLP\big(AvgPool(F)\big) + MLP\big(MaxPool(F)\big)\right)$$
$$= \sigma\left(W_1\left(W_0\big(F^c_{Avg}\big)\right) + W_1\big(W_0(F^c_{Max})\big)\right)$$

where $AvgPool(\cdot)$ and $MaxPool(\cdot)$ represent average pooling and maximum pooling respectively. W_1 and W_0 represent the weight values of the multi-layer perceptron respectively. $F^c_{Avg}\epsilon R^{1\times1\times c}$ represents the feature map after average pooling on the channel. $F^c_{Avg}\epsilon R^{1\times1\times c}$ represents the feature map after max pooling on the channel. $\sigma(\cdot)$ refers to Sigmoid gate function.

The spatial attention module can be defined as:

$$M_s(F) = \sigma\big(f^{7x7}([AvgPool(F); MaxPool(F)])\big)$$
$$= \sigma(f^{7x7}([F^S_{Avg}; F^S_{Max}]))$$

where $AvgPool(\cdot)$ and $MaxPool(\cdot)$ represent average pooling and maximum pooling respectively. $f^{7x7}(\cdot)$ represents the convolution operation of 7×7 . $F^s_{Avg}\epsilon R^{h\times w\times1}$ represents the feature map after spatial average pooling. $F^s_{Avg}\epsilon R^{h\times w\times1}$ represents the feature map after spatial max pooling. $\sigma(\cdot)$ refers to Sigmoid gate function.

Attention mechanism is an approach in deep network layer design where the goal is to recognize discriminative features in the inner activation maps and to utilize this knowledge toward enhanced task-specific data representation and improved model performance. This mechanism contributes to suppressing less relevant features and

emphasizing more important features for a considered task. At the level of TIL in assessing BC, important features lie in the location and shape of the tumor in the image.

The detailed structure diagrams of the five models are as follows:

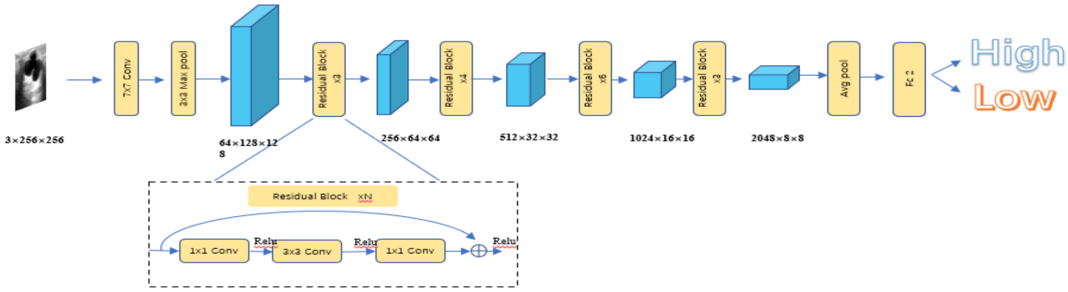

Fig. S1. Overall architecture of the ResNet50

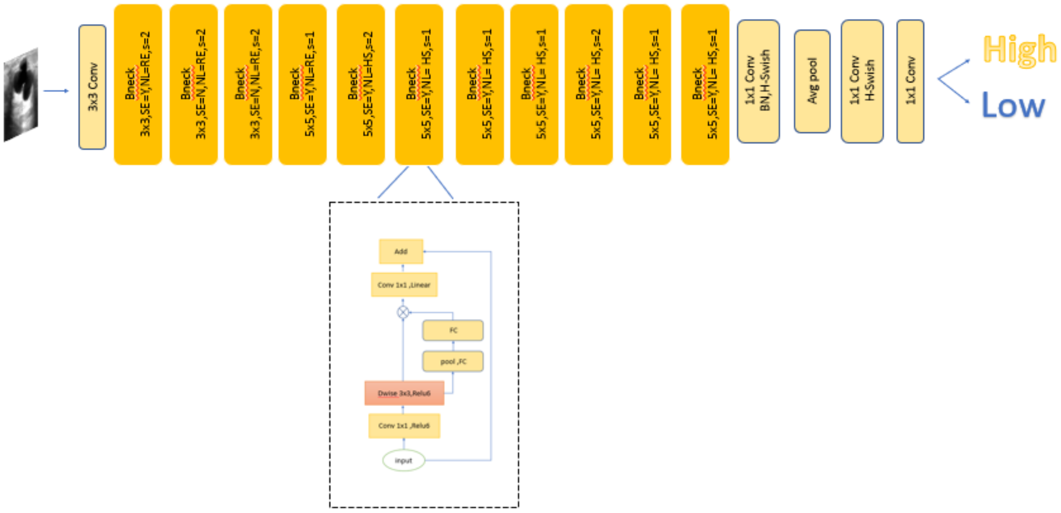

Fig. S2. Overall architecture of the MobileNet_v3 model

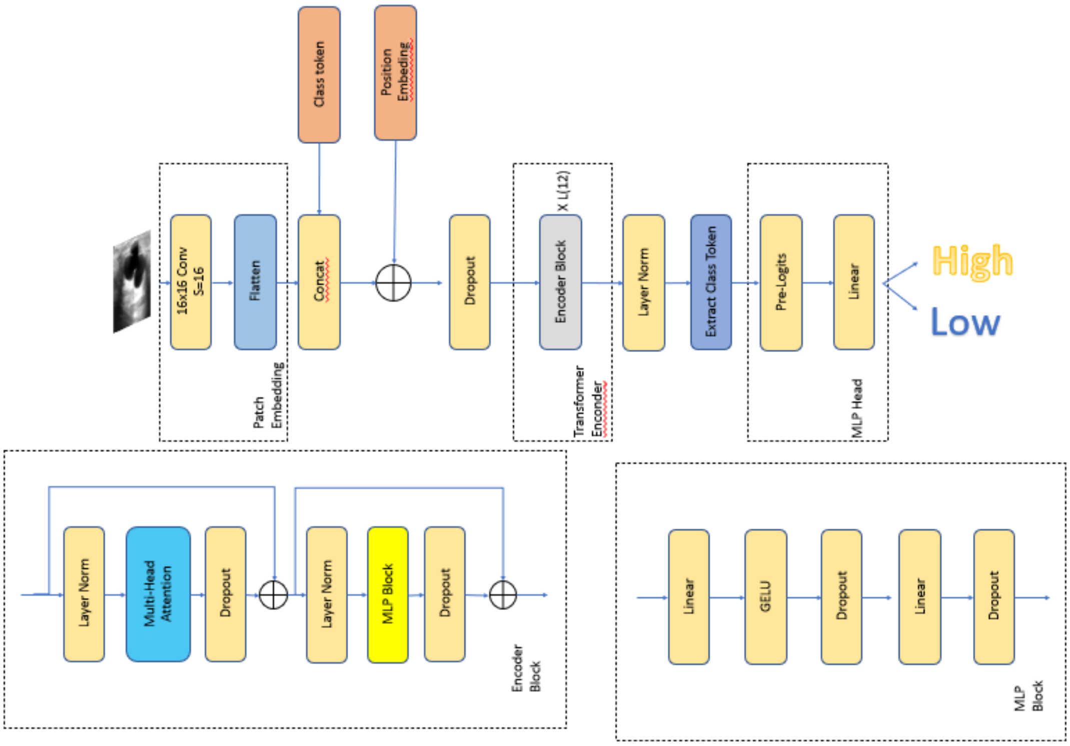

Fig. S3. Overall architecture of the Vision Transformer model

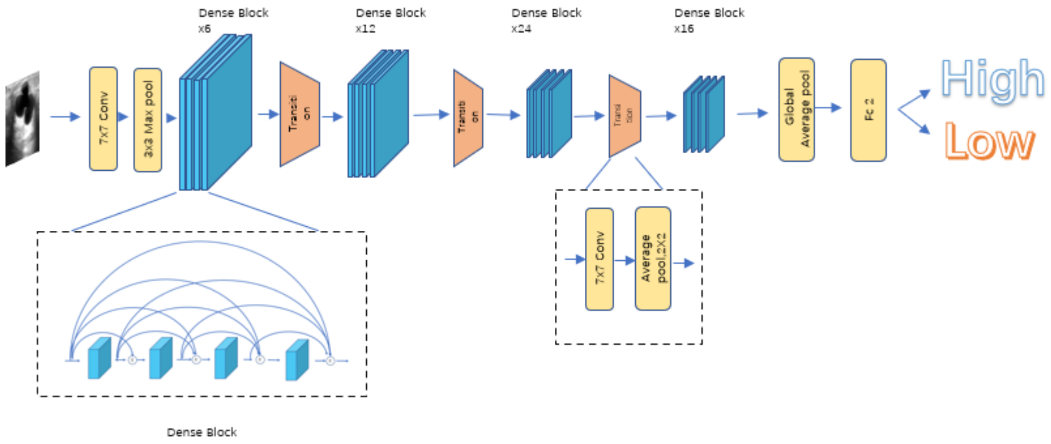

Fig. S4. Overall architecture of the DenseNet121 model

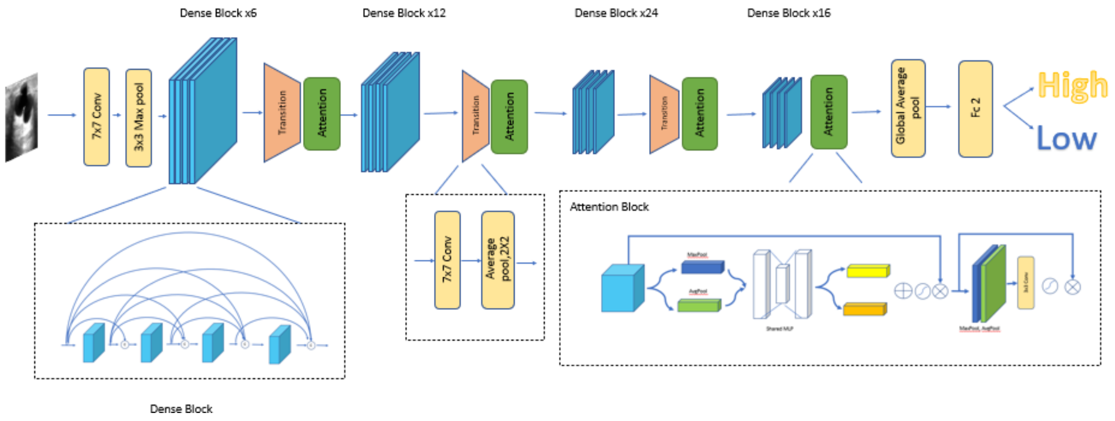

Fig. S5. Overall architecture of the attention-based DenseNet121 model

Methods S3. Strategy of training our model

In this study, supervised learning is employed to train our model. The loss function is expressed as follows:

$$loss(\mathrm{y}, \hat{y}) = -y\log(\hat{y}) + (1-y)\log(1-\hat{y})$$

where y denotes a true label, $\hat{y}$ denotes the corresponding prediction score. The cross-entropy loss function is the most commonly used loss function in classification, which is used to measure the difference between the distribution learned by the model and the real distribution. The smaller the cross-entropy value, the better the model prediction effect.

We adopted transfer learning strategy. we pretrained ResNet50, MobileNet_v3, Vision Transformer, DenseNet121 and attention-based DenseNet121, using 1.28 million natural images on the ImageNet dataset. The Adam[16] optimizer was used to optimize our model parameters, and the initial learning rate was set to 0.0001. When iterating to 40 epochs, select the model with the best AUC in the last 20 epochs. In order to improve the reliability of the network, each network is trained five times and chose the model with the median result for comparison. We trained the five models for 60 epochs on NVIDIA

GeForce GTX 3060 under Python 3.6 and Pytorch 1.9.0[17] deep-learning framework.

Methods S4. Measuring the performance of our model

In this study, we used the sensitivity, specificity, receiver operating curve (ROC)[18], area under curve (AUC), positive predict value (PPV), negative predict value (NPV)[19], and accuracy to evaluate the performance of the DL models. Let TP, TN, FP, and FN be the numbers of true positive, true negative, false positive, and false negative samples, respectively.

1. Sensitivity, specificity, receiver operating curve (ROC), and area under curve (AUC)

Sensitivity, also known as the true positive rate (TPR), reflects the ability to determine patients. Its mathematical formula is as follows:

$$Sensitivity = \frac{TP}{TP + FN}$$

Specificity, also known as the true negative rate (TNR), reflects the ability to determine non-patients. Its mathematical formula is as follows:

$$Specificity = \frac{TN}{TN + FP}$$

Positive predict value (PPV) reflects ability to misdiagnose. Its mathematical formula is as follows:

$$PPV = \frac{TP}{TP + FP}$$

negative predict value (NPV) reflects the ability to miss a detection. Its mathematical formula is as follows:

$$NPV = \frac{TN}{TN + FN}$$

Sensitivity, specificity, positive predict value (PPV),and negative predict value (NPV)

are determined under a certain threshold.

The receiver operating curve (ROC) reflects the trade-off between the sensitivity and specificity of the model for patient diagnosis. When drawing the ROC curve, the model prediction value of each patient was used as the threshold, and the sensitivity and specificity of the model results under these thresholds were calculated, and the ROC curve was drawn with 1-Specificity as the horizontal axis and sensitivity as the vertical axis. The area under the curve (AUC) was the area under ROC. The closer the AUC is to 1, the better is the model performance.

2. F1-score and Accuracy

The mathematical formula of F1-score[20] is

$$\mathrm{F1-score} = 2 * \frac{PPV * Sensitivity}{PPV + Sensitivity}$$

The larger F1-score, the better the model performance. At the same time, F1-score takes into account both the accuracy and recall of the classification model.

Accuracy reflects the percentage of all predictions that were correctly predicted. The calculation formula for the accuracy is

$$\mathrm{Accuracy} = \frac{TP + TN}{TP + FN + TN + FP}$$

Table S1. Details of the equipment used in each hospital

Research center	Ultrasound Diagnostic Instrument Information		
	Manufacturer	Model	Site
Hospital 1 (n= 396)	Philips (n = 328)	IU-22	Philips Medical Systems, Bothell, USA
		IU Elite	Philips Healthcare, Andover, MA, USA
	Siemens (n = 40)	ACUSON SEQUOIA 512	Siemens Medical Solutions, CA, USA
		ACUSON S2000	Siemens AG, Erlangen, Germany
	Others (n = 28)	Esaote MyLab60	Esaote Group, Italy
		TOSHIBA Aplio 300	Toshiba Medical Systems, Tokyo, Japan
Hospital 2 (n = 98)	Mindray (n = 69)	Resona 7	Mindray, Shenzhen, China
	Philips (n = 29)	IU-22	Philips Medical Systems, Bothell, USA

References

[1] Shorten C, Khoshgoftaar TM.A survey on image data augmentation for deep learning. *Journal of big data*2019;6:1-48.https://doi.org/

[2] Goodfellow I, Bengio Y, Courville A: **Convolutional networks**. In: *Deep learning. Volume 2016*, edn.: MIT Press Cambridge, MA, USA; 2016: 330-372.

[3] Glorot X, Bordes A, Bengio Y: **Deep sparse rectifier neural networks**. In: *Proceedings of the fourteenth international conference on artificial intelligence and statistics: 2011*: JMLR Workshop and Conference Proceedings; 2011: 315-323.

[4] Ioffe S, Szegedy C: **Batch normalization: Accelerating deep network training by reducing internal covariate shift**. In: *International conference on machine learning: 2015*: PMLR; 2015: 448-456.

[5] Lin M, Chen Q, Yan S.Network in network. *arXiv preprint arXiv:13124400*2013;

[6] LeCun Y, Bottou L, Bengio Y, Haffner P.Gradient-based learning applied to document recognition. *Proceedings of the IEEE*1998;86:2278-2324.https://doi.org/

[7] Bahdanau D, Cho K, Bengio Y.Neural machine translation by jointly learning to align and translate. *arXiv preprint arXiv:14090473*2014;

[8] Vaswani A, Shazeer N, Parmar N, Uszkoreit J, Jones L, Gomez AN, Kaiser Ł, Polosukhin I.Attention is all you need. *Advances in neural information processing systems*2017;30:

[9] He K, Zhang X, Ren S, Sun J: **Deep residual learning for image recognition**. In: *Proceedings of the IEEE conference on computer vision and pattern recognition: 2016*; 2016: 770-778.

[10] Simonyan K, Zisserman A.Very deep convolutional networks for large-scale image recognition. *arXiv preprint arXiv:14091556*2014;
[11] Howard A, Sandler M, Chu G, Chen L-C, Chen B, Tan M, Wang W, Zhu Y, Pang R, Vasudevan V: **Searching for mobilenetv3**. In: *Proceedings of the IEEE/CVF international conference on computer vision: 2019*; 2019: 1314-1324.
[12] Dosovitskiy A, Beyer L, Kolesnikov A, Weissenborn D, Zhai X, Unterthiner T, Dehghani M, Minderer M, Heigold G, Gelly S.An image is worth 16x16 words: Transformers for image recognition at scale. *arXiv preprint arXiv:201011929*2020;
[13] Huang G, Liu Z, Van Der Maaten L, Weinberger KQ: **Densely connected convolutional networks**. In: *Proceedings of the IEEE conference on computer vision and pattern recognition: 2017*; 2017: 4700-4708.
[14] Hu J, Shen L, Sun G: **Squeeze-and-excitation networks**. In: *Proceedings of the IEEE conference on computer vision and pattern recognition: 2018*; 2018: 7132-7141.
[15] Woo S, Park J, Lee J-Y, Kweon IS: **Cbam: Convolutional block attention module**. In: *Proceedings of the European conference on computer vision (ECCV): 2018*; 2018: 3-19.
[16] Kingma DP, Ba J.Adam: A method for stochastic optimization. *arXiv preprint arXiv:14126980*2014;
[17] Paszke A, Gross S, Chintala S, Chanan G, Yang E, DeVito Z, Lin Z, Desmaison A, Antiga L, Lerer A.Automatic differentiation in pytorch. 2017;
[18] Hajian-Tilaki K.Receiver operating characteristic (ROC) curve analysis for medical diagnostic test evaluation. *Caspian journal of internal medicine*2013;4:627.https://doi.org/
[19] Wong HB, Lim GH.Measures of diagnostic accuracy: sensitivity, specificity, PPV and NPV. *Proceedings of Singapore healthcare*2011;20:316-318.https://doi.org/
[20] Chicco D, Jurman G.The advantages of the Matthews correlation coefficient (MCC) over F1 score and accuracy in binary classification evaluation. *BMC genomics*2020;21:1-13.https://doi.org/
